# Comparative cost-efficiency of the EVOTECH endoscope cleaner and reprocessor versus manual cleaning plus automated endoscope reprocessing in a real-world Canadian hospital endoscopy setting

**DOI:** 10.1186/1471-230X-11-105

**Published:** 2011-10-03

**Authors:** Lindy Forte, Cynthia Shum

**Affiliations:** 1 VALORE Research, Toronto, Ontario, Canada; 2 Johnson and Johnson Medical Products, Department of Health Economics & Outcomes Research, Markham, Ontario, Canada

## Abstract

**Background:**

Reprocessing of endoscopes generally requires labour-intensive manual cleaning followed by high-level disinfection in an automated endoscope reprocessor (AER). EVOTECH Endoscope Cleaner and Reprocessor (ECR) is approved for fully automated cleaning and disinfection whereas AERs require manual cleaning prior to the high-level disinfection procedure. The purpose of this economic evaluation was to determine the cost-efficiency of the ECR versus AER methods of endoscopy reprocessing in an actual practice setting.

**Methods:**

A time and motion study was conducted at a Canadian hospital to collect data on the personnel resources and consumable supplies costs associated with the use of EVOTECH ECR versus manual cleaning followed by AER with Medivators DSD-201. Reprocessing of all endoscopes was observed and timed for both reprocessor types over three days. Laboratory staff members were interviewed regarding the consumption and cost of all disposable supplies and equipment. Exact Wilcoxon rank sum test was used for assessing differences in total cycle reprocessing time.

**Results:**

Endoscope reprocessing was significantly shorter with the ECR than with manual cleaning followed by AER. The differences in median time were 12.46 minutes per colonoscope (p < 0.0001), 6.31 minutes per gastroscope (p < 0.0001), and 5.66 minutes per bronchoscope (p = 0.0040). Almost 2 hours of direct labour time was saved daily with the ECR. The total per cycle cost of consumables and labour for maintenance was slightly higher for EVOTECH ECR versus manual cleaning followed by AER ($8.91 versus $8.31, respectively). Including the cost of direct labour time consumed in reprocessing scopes, the per cycle and annual costs of using the EVOTECH ECR was less than the cost of manual cleaning followed by AER disinfection ($11.50 versus $11.88).

**Conclusions:**

The EVOTECH ECR was more efficient and less costly to use for the reprocessing of endoscopes than manual cleaning followed by AER disinfection. Although the cost of consumable supplies required to reprocess endoscopes with EVOTECH ECR was slightly higher, the value of the labour time saved with EVOTECH ECR more than offset the additional consumables cost. The increased efficiency with EVOTECH ECR could lead to even further cost-savings by shifting endoscopy laboratory personnel responsibilities but further study is required.

## Background

Endoscopes are medical devices consisting of a long, thin, flexible or rigid tube equipped with a light and a video camera and are critical tools used in the screening and diagnosis of medical conditions such as cancer, breathing disorders, internal bleeding, and stomach ulcers. Endoscopes are also used to guide biopsies and laparoscopic surgery thereby avoiding the need for more invasive procedures. It has been estimated that about 17 million gastrointestinal endoscopic and 500, 000 flexible bronchoscopic procedures are performed each year in the United States [1,2].

During endoscopic procedures, the scopes come into contact with mucous membranes and bodily fluids and, therefore, must undergo thorough, reliable cleaning and high level disinfection between uses [3,4]. The cleaning and disinfection process requires meticulous, labor-intensive manual cleaning followed by high level disinfection. When conducted according to guidelines developed by the Society of Gastroenterology Nurses and Associates [3,4], manual cleaning is highly effective in reducing bioburden on endoscopes; failure to perform good manual cleaning can result in a failure in disinfection, increasing the risk of patient and staff exposure to microorganisms, including pathogens [5,6]. Because the results of this time-consuming process are dependent upon technique and method, outcomes can be variable [7,8]. With the need to reprocess endoscopes quickly to meet the demand in busy clinics, reprocessing personnel are under pressure to make the cleaning and disinfecting process as efficient as possible. Research has shown that manual cleaning practices vary from one facility to another whereby one survey showed that only 43% of centers were fully compliant with national guidelines [7]. As a result, the SGNA states that, "inadequate cleaning of endoscopes has been one factor cited in transmission of infection by flexible endoscopes" [3]. There are also reports in the literature of patient-to-patient transmission of serious infection following inadequate cleaning of endoscopes [9,10].

The need for a more automated, reliable cleaning and disinfection process led to the development of the EVOTECH^® ^Endoscope Cleaner and Reprocessor (ECR) which was the first system to receive U.S. Food and Drug Administration approval to eliminate manual pre-cleaning of the endoscope prior to its automated high-level disinfection processing [11]. The effectiveness of the EVOTECH ECR was demonstrated to be 99% to 100% effective in meeting or surpassing the cleaning endpoints set for protein, hemoglobin and bioburden residuals in a recent study of actual clinic use and simulated use [12]. The authors of the efficacy study concluded that the EVOTECH ECR is an effective automated approach that ensures surfaces and channels of flexible endoscopes are adequately cleaned after having only a bedside pre-cleaning consisting of a flush with enzymatic detergent solution through all channels and wiping of the exterior of the insertion tube using a cloth moistened with the same enzymatic detergent. With EVOTECH ECR, manual cleaning is not required except in cases where reprocessing is delayed for more than one hour or in emergency situations where patients are not properly prepped prior to the procedure. Based on this research, the FDA concluded that manual cleaning of endoscopes is not required prior to processing in the EVOTECH ECR as long as a cycle including a wash stage is selected. The wash stage completed in the EVOTECH ECR eliminates tedious manual brushing of the endoscopes and results in cleaning that is safe, fast and consistent each time.

While it appears that the EVOTECH ECR would save valuable time and effort on the part of staff responsible for reprocessing endoscopes, there are no studies quantifying the total time required to reprocess endoscopes with EVOTECH ECR compared to manual cleaning plus AER disinfection. The purpose of this economic evaluation was to determine the cost-efficiency of using the EVOTECH ECR versus an AER disinfection process from the perspective of hospital decision makers managing operating budgets by comparing the annual utilization costs, including labour and consumable supplies, involved in using each reprocessor type in an actual clinical practice setting in Canada.

## Methods

### Time and Motion Study

A time and motion study was conducted at a Canadian hospital to collect data on the personnel resources and consumable supplies costs associated with the use of EVOTECH^® ^ECR (Advanced Sterilization Products (ASP), a unit of Johnson & Johnson Medical Products) and Medivators^® ^Reprocessing Systems (DSD-201 Automated Endoscope Reprocessor (AER); Minntech Corporation). Following a pre-study visit where the disinfection process was observed, a data collection form was developed to objectively capture the time involved in each stage of the reprocessing procedure and to quantify the disposable equipment supplies consumed. The reprocessing of all colono-, gastro-, and broncho-scopes by two technicians in the endoscopy unit were then observed over three days during two visits. Three stopwatches were used to simultaneously collect all scope reprocessing times as outlined in Table 1. A wet leak test was not routinely performed for endoscopes to be reprocessed with the EVOTECH ECR because the EVOTECH ECR performs automated wet and dry leak tests. Because some sites may choose to perform the manual wet leak test in addition to the leak testing in EVOTECH ECR, the time required for the manual wet leak was incorporated in the total per scope labour times for EVOTECH ECR.

**Table 1:** Steps observed in the reprocessing of endoscopes

	EVOTECH ECR	Manual cleaning plus AER*
Phase 1 - Preparation of scopes for reprocessing*		

Fill sink with water	x	x

Clean valves *Remove suction, air, water and biopsy valves in sink; brush the valves and other removable parts; place valve parts in the reprocessor*.	x	x

Manual wet leak test**	x	x

Add enzymatic detergent to sink		x

Brush internal channels three times each		x

Connect cleaning adapters/tubing to scope		x

Flush interior of scope with enzymatic detergent using hospital-developed automated flushing system		x

Drain sink of enzymatic detergent		x

Rinse interior of scope with tap water using hospital-developed automated flushing system		x

Rinse exterior of scope with tap water spray		x

Drain sink and remove scope from sink	x	x

Dry exterior of scope with a blue wipe		x

Walk from sink to reprocessor with scope	x	x

Place scope in an unoccupied basin of reprocessor	x	x

Connect colour coded connecting tubing to scope according to specific scope connection diagram	x	

Enter identification data for operator, physician, scope type, procedure and patient***	x	

Connect reprocessing adapter/tubing to connection port in basin		x

Place lid closure on reprocessor	x	x

Program and start cycle	x	x

**Phase 2 - Removal of scopes from reprocessor at conclusion of cycle^#^**		

Enter operator identification	x	x

Open reprocessor lid	x	x

Disconnect color coded tubing from scope	x	

Disconnect reprocessing adapter/tubing from connection port in basin with manual check to ensure connections were properly placed		x

Return accessories and scope to Cleanascope^® ^transport tray for next procedure or to the storage cupboard if the scope is not required in the short term	x	x

*Included all steps involved in preparing scope for reprocessing and then starting the reprocessing cycle.

**The EVOTECH ECR performs an automated wet and dry leak test. A manual leak test according to the endoscope manufacturer's instructions is also recommended. Thus, although it may not be required with EVOTECH ECR, a manual wet leak test was incorporated in the analysis.

***Although the Medivators DSD-201 was capable of recording the same information, this procedure was not in place at the study site.

^#^Included all steps required to remove scope from reprocessor and replace scope in storage.

Timing was paused during the data collection period if a technician was distracted by conversation such that the normal activity was stopped completely (i.e., if a technician was asked to leave the endoscope reprocessing activity to attend to something else); otherwise, normal conversation and the negative impact that this would have on a technician's speed and efficiency was included for both the EVOTECH ECR and the AER cycles equally. The impact of technicians' efficiencies during the manual cleaning step (required with the AER only) was incorporated when it occurred. For example, at the study site, a semi-automated cleaning process was in place whereby, following a wet leak testing and manual brushing of the endoscope channels, a machine developed by engineers at the hospital was connected to the endoscope to complete the enzymatic and tap water flushes. Efficiencies in the process occurred when technicians were able to initiate the draining of the sink before the enzymatic flushing process was fully complete such that the tap water rinse could start immediately without the need to wait for the sink to drain.

After each scope was placed in a separate basin of each reprocessor type, the actual durations of the various phases of the reprocessing period (i.e., the leak test, pre-rinse, wash, rinse, disinfection, final rinse and alcohol flush stages for EVOTECH ECR and the disinfection, rinse, alcohol flush and air purge for the AER) were not timed because it was not practical to do so. With the requirement to concurrently monitor all of the activities of two laboratory technicians simultaneously employing a total of up to 8 reprocessing bays, it was determined that there would be too much error introduced if more than three stopwatches requiring different start, stop, and re-start times were used. Instead, for the EVOTECH ECR, an average cycle duration was calculated based on the read-outs produced following each cycle. According to the EVOTECH ECR Technical Manual [13], individual cycle durations can be between 30 and 33 minutes. The average cycle duration for the EVOTECH ECR cycles observed during the study was 31 minutes. The high-level disinfection phase was 5 minutes 20 seconds at 50°C followed by two 45 second tap water rinses at 35°C. With the Medivators DSD-201 AER, there was a variance in cycle time depending upon the scope type. The total cycle lengths displayed on the unit were assumed as follows: colonoscope 40 minutes; gastroscope 35 minutes; bronchoscope 35 minutes. According to the clinic's practice, the AER was programmed to soak all scopes in high-level disinfectant for 5 minutes at 28-29°C. The total reprocessing time for colonoscopes was 5 minutes longer because the AER was programmed to perform two tap water rinses.

The time involved in all procedures that were common to both reprocessor types (e.g., gross pre-cleaning in operatory rooms, walk from operatory to endoscope reprocessing laboratory, disposal of sterile tray liner, cleaning of endoscope trays, preparation of endoscope trays for next procedure) was not collected as the process was the same for both reprocessor types.

### Calculation of Scope Processing Times

The total time taken for reprocessing was calculated by adding the observed time for the manual cleaning, leak testing, standard reprocessing time for each scope type, and removal of scopes from the reprocessors. An average time for each type of scope reprocessed with the ECR versus manual cleaning plus AER was calculated by adding the total time separately for all colono-, gastro-, and broncho-scopes for each reprocessor and then dividing the total by the number of each type of scope. The average time per scope was calculated without and with the standard cycle reprocessing times to obtain the time for the personnel labour only and the total time including automated high-level disinfection.

The total number of scopes processed annually by the hospital in the Sterile Processing Department was available for the most recent full year. From April 1, 2008 to March 31, 2009, the endoscopy unit at L'Hôpital Maisonneuve-Rosemont reprocessed 5, 780 colonoscopes, 3, 550 gastroscopes, and 1, 058 bronchoscopes. A "typical day" was calculated by dividing the total number of each scope type by 260 clinic days per year. The number of minutes taken in the reprocessing of all scopes for a typical day was calculated by multiplying the number of each scope type by the observed average number of minutes per scope. Savings in time related to technician labour and reprocessing cycle time were calculated by subtracting the total time for a "typical day" using only manual cleaning plus AER from the total time for a typical day using only the EVOTECH ECR.

### Value of the Time Saved

The scope reprocessing time saved was multiplied by a technician wage of CDN$22.20 per hour including benefits (Human Resources Department, L'Hôpital Maisonneuve-Rosemont).

### Statistical Analysis

A scope reprocessing cycle was considered evaluable for the analysis if both phases of the reprocessing (i.e., manual cleaning/leak testing and removal from the reprocessor) were captured. In some cases, due to the timing of lunch breaks or the start of the cycle occurring too late in the day, the first or second phase of the cycle was not timed and, therefore, the cycle was excluded from the analysis. Endoscopic Retrograde Cholangiopancreatography (ERCP) scopes were also excluded because there were too few processed (2 in the ECR and 1 in the AER) during the timeframe of the study. SAS version 9.2 (SAS Institute, Cary, NC) was used for the statistical analysis. Exact Wilcoxon rank sum test was used for assessing the difference in total time spent on colono-, gastro-, and bronchoscopes between EVOTECH ECR and manual cleaning plus AER. Data were presented as median followed by interquartile range. For all the analyses, p < 0.05 was considered as statistically significant. All cost figures are reported in Canadian dollars.

## Results

The total numbers of each scope type that were evaluable for the analysis are shown in Table 2. One cycle of each reprocessor type was excluded because the cycles had to be repeated due to human error in connecting the tubing. For the EVOTECH ECR, the cycle was automatically interrupted after 13 minutes due to a detected disconnection in an endoscope connector. The connection was fixed and the cycle was repeated. The lost time was, therefore, between 14-15 minutes. For the AER, a similar poor connection was discovered by the user but only after the completion of the cycle because the AER did not have an alarm or other signal to indicate when the tubing disconnected from the scope. After the poor connection was discovered, the connection was repaired and the cycle was restarted. The lost processing time was estimated at 35.5 minutes (35 minutes to repeat the cycle plus 0.5 minutes to open the reprocessor and discover and fix the improper connection). In addition, a total of 14 (14%) cycles were excluded from the analysis because, due to the timing of staff breaks, either the first or second phase of the cycle could not be timed.

**Table 2:** Mean total reprocessing time and time savings with the EVOTECH ECR

	Colonoscope	Gastroscope	Bronchoscope
**EVOTECH ECR**			
Number of scopes	21	9	4
Median time (mins) per scope	37.42	37.22	36.04
[inter-quartile range]	[36.72, 37.63]	[36.65, 37.38]	[35.69, 36.58]
			
**Manual cleaning plus AER**			
Number of scopes	23	16	8
Median time (mins) per scope	49.88	44.27	42.43
[inter-quartile range]	[49.42, 51.12]	[42.71, 45.37]	[41.70, 43.81]
			
**Difference between EVOTECH ECR and Manual cleaning plus AER (mins)**	**12.46**	**6.31**	**5.66**
p-value*	< 0.0001	< 0.0001	0.0040

*Wilcoxon rank sum test

### Time Consumed in Reprocessing Scopes

The total time to reprocess all scope types was significantly shorter in the EVOTECH ECR compared with manual cleaning plus AER. The differences in median time to reprocess each scope in the EVOTECH ECR versus manual cleaning plus AER were 12.46 minutes per colonoscope (p < 0.0001), 6.31 minutes per gastroscope (p < 0.0001), and 5.66 minutes per bronchoscope (p = 0.0040). The average total number of minutes required to reprocess each type of scope along with the time difference between the reprocessor types are shown in Table 2.

The total labour time spent on each scope type was also similarly shorter for the EVOTECH ECR (Table 3). The differences in median labour time to reprocess each scope in the EVOTECH ECR versus manual cleaning plus AER were 2.61 minutes for each colonoscope (p < 0.0001), 2.20 minutes for each gastroscope (p = 0.00002), and 1.54 minutes for each bronchoscope (p = 0.0485). Cycle reprocessing with EVOTECH ECR required between 20% to 30% less time than manual cleaning plus AER despite the fact that a large portion (approximately 2-3 minutes per cycle) of the technician's labour was spent entering the identification information for the operator, physician, and patient (data not shown) with EVOTECH ECR. These activities were not performed with the AER although a technical manual for Medivators DSD-201 indicates that this information can be collected [14].

**Table 3:** Labour only time involved in reprocessing with each reprocessor type

	Colonoscope	Gastroscope	Bronchoscope
**EVOTECH ECR**			
Median time (mins) per scope	7.27	7.07	5.89
[interquartile range]	[6.57, 7.48]	[6.50, 7.23]	[5.54, 6.44]
**Manual cleaning plus AER**			
Median time (mins) per scope	9.88	9.27	7.43
[interquartile range]	[9.42, 11.12]	[7.71, 10.37]	[6.70, 8.81]
**Difference between EVOTECH ECR and Manual cleaning plus AER (mins)**	**2.61**	**2.20**	**1.54**
p-value*	< 0.0001	0.00002	0.0485

*Wilcoxon rank sum test

### Time Savings with EVOTECH ECR

The daily savings in labour and total reprocessing time are presented in Table 4. The time savings achieved on a daily basis with the EVOTECH ECR were 6.2 hours for total cycle time and 1.8 hours of direct labour time.

**Table 4:** Average daily total reprocessing and personnel labour times

	Colonoscopes	Gastroscopes	Bronchoscopes
Typical average number of scopes per day	22	14	4
***Total reprocessing time***			
**EVOTECH ECR**			
Average time per scope (mins)	38.22	37.92	36.99
Total daily reprocessing time (mins)	839	518	151
**Manual cleaning plus AER**			
Average time per scope (mins)	50.29	44.24	42.64
Total daily reprocessing time (mins)	1104	604	174
**Difference between EVOTECH ECR and Manual cleaning plus AER**			
Savings in total reprocessing time with EVOTECH ECR (mins)	265	86	23
Total daily time savings for reprocessing all scopes	**374 minutes; 6.2 hours**		
***Labour only time***			
**EVOTECH ECR**			
Average time per scope (mins)	7.22	6.92	5.99
Total daily labour time (mins)	159	95	24
**Manual cleaning plus AER**			
Average time per scope (mins)	10.29	9.24	7.64
Total daily labour time (mins)	226	126	31
**Difference between EVOTECH ECR and Manual cleaning plus AER**			
Savings in total reprocessing time with EVOTECH ECR (mins)	**67**	**32**	**7**
Total daily time savings for reprocessing all scopes	**106 minutes; 1.8 hours**		

### Labour Cost Savings with EVOTECH ECR

The value of the total time saved every day with EVOTECH ECR (6.2 hours) was $138.41 per day and $35, 987 per year. The value of the direct labour time saved every day with EVOTECH ECR (1.8 hours) was $39.07 per day and $10, 159 per year.

### Consumables and Additional Hospital Personnel Time Utilized in Reprocessing

Tables 5 and 6 show all of the consumables used in reprocessing scopes and the additional labour involved in equipment maintenance for the AER and the EVOTECH ECR, respectively.

**Table 5:** Cost of consumables involved in reprocessing endoscopes with manual cleaning plus AER

	Cost per Scope	Cost per Year
Multiuse brushes	$1.11 (broncho) $1.59 (colono/gastro)	$15, 860.21*
Sterilization of multiuse brushes	$0.14	$1, 443.00
Test strips	$1.21	$12, 465.17
Fibertech Enzymatic soap (4L)	$0.70	$7, 207.53
Cidex OPA** in AER for automated endoscope cleaning	$4.15	$42, 822.86
Labour to change Cidex after every 45 cycles	$0.09	$933.02
70% alcohol for alcohol flush	$0.006	$62.05
Cidex OPA in ultrasonic device to clean multiuse brushes	$0.03	$284.58
Blue wipes to dry endoscopes after manual cleaning	$0.29	$3, 037.03
Blue wraps to cover brushes to be autoclaved at the end of each day	$0.004	$38.27
Small filter (6 per year)	$0.009	$90.00
Ultra filter replacement every 2 years	$0.04	$425.00
Ultra filter cleaning 3 times per year - labour	$0.03	$360.00
Ultra filter cleaning 3 times per year - Cidex	$0.07	$700.50
**Total annual consumables cost**		**$85, 729.23**
**Cost of consumables per scope**		**$8.31**

*Annual cost of disposable brushes would be much higher at $82, 528.

**A less costly disinfectant such as glutaraldehyde may be used; if glutaraldehyde was used, the disinfectant cost would be lower.

**Table 6:** Cost of consumables involved in reprocessing endoscopes in EVOTECH ECR

	Cost per Scope	Cost per Year
Cidex OPA for automated, single-use cleaning of each endoscope	$7.75	$79, 912.68
Labour to change the Cidex	$0.02	$168.10
70% alcohol for internal flush of each endoscope	$0.03	$310.27

Cidezyme for automated daily disinfection of the EVOTECH ECR	$0.89	$9, 174.31
Labour to perform auto disinfection cycle	$0.003	$28.86
1 μm External Pre Filter	$0.09	$225.00
0.2 μm Internal Evotech Machine Filter	$0.33	$825.00
0.2 μm External Hybrid Carbon Filter	$0.16	$400.00
0.2 μm External Membrane Filter	$0.32	$800.00
Labour to change filters	$0.02	$49.95
**Total annual consumables cost**		**$91, 894.17**
**Cost of consumables per scope**		**$8.91**

### Total per Cycle Cost Savings with EVOTECH ECR

The total per cycle cost of consumables and labour for maintenance was slightly higher for EVOTECH ECR versus the AER ($8.91 versus $8.31, respectively). Including the cost of labour consumed in reprocessing scopes, the per cycle and annual costs of using the EVOTECH ECR was less than the cost of using the AER ($11.50 versus $11.88) as shown in Table 7. With a cost savings of $0.38, it was estimated that the hospital would save $3, 920 per year to reprocess the 10, 316 endoscopes used annually.

**Table 7:** Total per cycle and annual costs of labour plus consumables

	EVOTECH ECR	Manual cleaning plus AER
Annual cost of consumables	$91, 894.17	$85, 729.23
Annual cost of labour*	$26, 695.03	$36, 854.22
Total annual cost**	$118, 589.20	$122, 583.45
**Total cost per cycle**	**$11.50**	**$11.88**

*Does not include personnel labour time elapsed during cycle processing as employees could be available to work on other tasks while a scope is in the reprocessor.

**Does not include acquisition cost of either processor

### Efficiency

With shorter cycles in EVOTECH ECR, the total per scope time saved would be 374 minutes per day. With a weighted average total reprocessing time of 38 minutes per scope for all scope types processed in EVOTECH ECR, an additional 9.84 scopes could be processed every day and 2, 558 additional scopes could be processed each year. This represents an increase of 25% additional scopes each day (9.84/40).

## Discussion

This study examining the cost-efficiency of EVOTECH ECR versus manual cleaning plus AER captured the time-consuming and labor-intensive efforts required for the critical cleaning and disinfection of endoscopes during actual gastroenterology clinic practice in a high-volume Canadian hospital. The investigation demonstrated that a substantial amount of clinic personnel time is saved on a daily basis when the EVOTECH ECR is used rather than a reprocessor that requires manual cleaning of endoscopes. For each endoscope processed, EVOTECH ECR saved a total of 12.5 minutes per colonoscope, 6.3 minutes per gastroscope, and 5.7 minutes per bronchoscope compared with manual cleaning plus AER. The significant per cycle time savings was seen despite the 2-3 minutes required every cycle for the technician to enter identification information for the operator, procedure, physician and patient, a feature that was not employed with the AER in use in the study clinic. Had this feature been activated, an even greater time difference between the EVOTECH ECR and the AER reprocessing cycles could be expected.

Over the course of a day, the per scope time savings translated into a savings of 6.2 hours of time spent in the reprocessing laboratory and almost 2 hours of technician direct labour time alone. The analysis showed that the value of the time saved would be about $35, 987 per year in a technician's salary; alternatively, 25% more scopes could be reprocessed annually at the same labour cost. While the cost of consumable supplies required for endoscope processing and reprocessor maintenance was slightly higher with EVOTECH ECR ($8.91 vs. $8.31 per scope), the reduced labour cost more than offset the higher cost of consumed and disposable supplies such that the total per scope costs were $11.50 with EVOTECH ECR and $11.88 with manual cleaning plus AER.

On average, endoscopes are used up to 1200 times annually [14]. Great care must be taken during cleaning because they are fragile and expensive to replace. There must also be certainty that infectious agents are destroyed to prevent cross-contamination from one patient to another. Thus, the demand for rapid turnover of endoscopes must be balanced with the need to ensure patient health. Despite the availability of state of the art equipment, highly technical cleaning solutions and detergents, and guidelines for the Standards of Infection Control in Reprocessing of Flexible Gastrointestinal Endoscopes [4], human error has led to the potential spread of infection through endoscopes that weren't adequately cleaned [9,10,15-17].

Recent surveys report that manual cleaning is inadequately performed at least some of the time [7,8,18]. Other published reports suggest that the time spent and steps taken to manually clean flexible endoscopes in clinic practice are highly variable and can range from 4 to 25 minutes [19]. Moreover, the time taken to manually clean endoscopes according to guidelines may be up to five fold higher than the time taken spent manually cleaning endoscopes in usual clinic practice [19]. To achieve reliable, consistent results and minimize the chance of cross-contamination, manufacturer and SGNA guidelines must be meticulously followed. By removing the manual cleaning step, the EVOTECH ECR's automated cleaning process can not only minimize the likelihood of human error but also increase clinic efficiency and decrease overall costs.

This study was the first to directly compare the efficiency and cost of reprocessing endoscopes with EVOTECH ECR versus manual cleaning and reprocessing with an AER in an actual Canadian clinic practice. Strengths include the fact that the study was designed from the perspective of hospital administrators responsible for operating budgets interested in the costs and benefits of using a newer reprocessor relative to their existing equipment; as such, actual clinic procedures were recorded by an unbiased, third-party observer and actual clinic methods and costs were incorporated.

However, it should be noted that the study did not examine differences in acquisition and maintenance contract costs of EVOTECH ECR vs. AERs which may be important for capital purchasing departments. The relative acquisition cost of the two reprocessor types could not be included because comparative pricing was not publicly available in Canada.

Another strength of the study lies in the design of the investigation where bias against manual cleaning plus AER was avoided for three main reasons. First, the time required to conduct a manual leak test of all scopes to be reprocessed in the EVOTECH ECR as per the EVOTECH user manual was included. Because the EVOTECH ECR conducts an automated wet and dry leak test, some users may not perform a manual leak test as per endoscope manufacturers' instructions. To be conservative, the time required to perform a wet leak test was included in the EVOTECH ECR data collection even though a recent study has shown that the manual leak test may not be required [12]. Second, the hospital selected for the comparison of the reprocessors was particularly efficient in the manual cleaning process due to the use of an in-house developed, non-commercial flushing device. The device conducted the enzymatic and water flushes of the endoscope channels through an automated process without the need for clinic personnel to change hosing and/or attach and remove sources of detergent or water as required. Third, all of the recommended steps for reprocessing of endoscopes with EVOTECH ECR were timed including the entry of operator, physician and patient identification for every cycle; there was no recording of this information in the AER.

A limitation of the study is the fact that an evaluation of the effectiveness of reprocessing scopes in EVOTECH ECR versus manual cleaning plus AER could not be incorporated in conjunction with the time and motion study. However, other published references have demonstrated that cleaning in the EVOTECH ECR is non-inferior to manual cleaning using worst-case conditions for the EVOTECH ECR and best-case conditions for manual cleaning and according to standards set in consultation with the FDA based on published literature [13]. In addition, a recent Canadian publication showed that the use of EVOTECH ECR to clean endoscopes in actual practice met or exceeded standards set for routine manual cleaning in at least 99% to 100% of cleaning cycles [12]. Combined, the research suggests that EVOTECH ECR's automated process is at least as effective as manual cleaning plus AER and, when the value of human labour involved in the cleaning and reprocessing procedures is included, use of EVOTECH ECR is also cost-saving.

Within the scope of this study, it was not possible to determine whether EVOTECH ECR would be more efficient and less costly to use than all other AERs on the Canadian market nor was it possible to determine if EVOTECH ECR would be less costly in countries outside of Canada. It should also be noted that there are some cases in which automated cleaning in EVOTECH ECR is not possible. For example, EVOTECH ECR cannot be used to reprocess double biopsy channel scopes and ultrasound scopes. In addition, in cases where endoscopes are used for emergency procedures or where reprocessing is delayed for more than one hour, manual cleaning of the endoscopes to be reprocessed with EVOTECH ECR is still required [12]. Therefore, in these relatively infrequent scenarios, the cost of reprocessing in the EVOTECH ECR could be higher than the cost with manual cleaning plus AER because the labour cost savings associated with the avoidance of manual cleaning would not be realized. Future research could be conducted over a time period long enough to ensure that the rare incidences where manual cleaning of endoscopes to be reprocessed with EVOTECH ECR could be included in order to examine the impact that the additional cleaning time would have on the overall cost comparison of EVOTECH ECR vs. an AER.

The present study was designed to determine efficiency and cost on a micro or per cycle basis while not interrupting the usual clinic practice where both reprocessor types were used continuously each day. Consequently, the ability of the study to project overall costs or savings to the endoscopy department with the use of EVOTECH ECR versus manual cleaning plus AER on a more macro level was limited. Future research could monitor the use of EVOTECH ECR compared with manual cleaning and reprocessing with an AER by repeating the study on one day with the use of EVOTECH ECR only and another day with an AER only. A study of this design would allow researchers to determine the full length of time used to clean and disinfect an equivalent number of scopes taking into consideration the fact that each of the reprocessors can clean two endoscopes concurrently and, thus, the difference in total reprocessing time required with the two reprocessor types would be less than the 6.2 hours reported in this study. The clinic set-up at this study site, where both reprocessor types were used continuously each day to keep up with the demand for clean endoscopes, did not permit a comparison on this macro level. In addition, with a macro level examination of endoscopy clinic efficiency comparing different reprocessors, such a study could determine staff's ability to perform other clinical duties while still ensuring efficient reprocessing of endoscopes. Finally, the full impact of failed cycles could be incorporated where, if the EVOTECH ECR fails during a cycle because a connection was not made properly, the computer notifies the technician right away with a visual and auditory signal along with the reason for the failed cycle. With the immediate notification, the connection can be rectified and the cycle can be re-started. The AER in use at this study site did not have a signal to notify the operator of a poor connection. The impact of these additional efficiencies or safety features with EVOTECH ECR compared with an AER could not be incorporated into the present study, but should be considered in future research.

The present study was also limited in its ability to examine the impact of other potential advantages of the EVOTECH ECR over AERs. First, the long term use of the EVOTECH ECR rather than an AER may reduce the cost related to the number of scopes required annually. With wear and tear on endoscopes associated with manual brushing [20,21], scopes may have to be replaced and/or repaired more frequently. Second, the EVOTECH ECR records information about the doctor, patient, scope type, and operator allowing improved tracking of information in case of a system failure. Third, there is less environmental impact with the EVOTECH ECR because an AER requires the use of more disposable supplies in the manual cleaning step.

While this study predicts significant savings in terms of human labour, it is unknown whether, in practice, those savings could be redistributed to other functions within the clinic or hospital or whether 25% more scopes could actually be reprocessed in a day without the need to expand the physical structure of the laboratory. Future research could examine precisely how the time and cost savings with the use of EVOTECH ECR could be realized through shifting personnel responsibilities. For example, at the study site, personnel in the endoscopy reprocessing unit utilize any downtime during the automated portion of the reprocessing cycle to assist other staff to prepare beds and the operatories where the endoscopy procedures take place. It is possible that the total number of personnel employed in the gastroenterology clinic could be reduced through efficiencies like these.

## Conclusions

In summary, the EVOTECH ECR was more efficient and less costly to use for the reprocessing of endoscopes than manual cleaning plus AER as shown in actual practice in a busy endoscopy unit in Canada. Although the cost of consumable supplies required to reprocess endoscopes with EVOTECH ECR was slightly higher than with manual cleaning plus AER, the value of the significantly shorter labour time with EVOTECH ECR more than offset the additional consumables cost. Further research should be done to determine if the increased efficiency with EVOTECH ECR would permit endoscopy clinics to realize even further cost-savings by shifting endoscopy laboratory personnel responsibilities.

## Competing interests

CS is an employee of Johnson and Johnson Medical Products. LF is an employee of VALORE Research. VALORE Research received funding (including the article-processing charge) for the study provided by Johnson and Johnson Medical Products, a division of Johnson and Johnson Canada. VALORE Research had independent control over the methods of the study and had the right to publish the analysis regardless of its results. VALORE Research will not gain or lose financially from the publication of this manuscript.

## Authors' contributions

All authors have read and approved the final manuscript. CS was responsible for the study concept and provided direction for the conduct of the data collection. LF designed the study and data collection forms, performed all data collection, interpreted the data, and wrote the manuscript with input from CS. LF had independent control over the study methods and collection of the data. Advanced Sterilization Products personnel provided assistance with the editing of the manuscript.

## Pre-publication history

The pre-publication history for this paper can be accessed here:

http://www.biomedcentral.com/1471-230X/11/105/prepub
